# Memory CD4^+^ T Cells in Immunity and Autoimmune Diseases

**DOI:** 10.3390/cells9030531

**Published:** 2020-02-25

**Authors:** Itay Raphael, Rachel R. Joern, Thomas G. Forsthuber

**Affiliations:** 1 Department of Neurological Surgery, University of Pittsburgh, UPMC Children’s Hospital, Pittsburgh, PA 15224, USA; 2 Department of Biology, University of Texas at San Antonio, San Antonio, TX 78249, USA; Rachel.Joern@utsa.edu

**Keywords:** CD4^+^ T cells, memory T cells, autoimmune disease, effector memory T cell, central memory T cell, tissue-resident T cell

## Abstract

CD4^+^ T helper (Th) cells play central roles in immunity in health and disease. While much is known about the effector function of Th cells in combating pathogens and promoting autoimmune diseases, the roles and biology of memory CD4^+^ Th cells are complex and less well understood. In human autoimmune diseases such as multiple sclerosis (MS), there is a critical need to better understand the function and biology of memory T cells. In this review article we summarize current concepts in the field of CD4^+^ T cell memory, including natural history, developmental pathways, subsets, and functions. Furthermore, we discuss advancements in the field of the newly-described CD4^+^ tissue-resident memory T cells and of CD4^+^ memory T cells in autoimmune diseases, two major areas of important unresolved questions in need of answering to advance new vaccine design and development of novel treatments for CD4^+^ T cell-mediated autoimmune diseases.

## 1. Introduction

CD4^+^ T helper (Th) cells play a central role in the immune system and carry out multiple functions including activation, coordination, modulation, and regulation of innate and adaptive immune responses. These various functions of Th cells are necessary to attain effective immune responses against a variety of different pathogens, while maintaining self-tolerance and avoiding undesired attacks against self-tissues [1,2,3]. The regulation of immune responses by Th cells is accomplished through the secretion of specific cytokines, which, together with a “master” regulatory transcription factor, define the respective Th cell subset and its specialized functions and attributes [1,2,3,4]. While much is known about the effector function of Th cells, the roles and biology of memory CD4^+^ T cells are more complex and less well understood. Moreover, the function of memory CD4^+^ T cells in mounting an immune response can only partially be defined by the precursor Th subset from which the primary immune response originated. An additional layer of complexity is added by the different memory CD4^+^ T cell subsets generated during the primary immune response [3,5]. Memory T cells are generally subdivided into three main populations: central memory T cells (T_CM_), effector memory T cells (T_EM_), and tissue-resident memory T cells (T_RM_). At present, these memory T cell subsets are primarily characterized by their phenotype, migratory properties, and tissue homing patterns, which in many instances imply unique functional attributes [5,6,7].

Antigen (Ag)-specific naïve CD4^+^ T cells are first activated in lymphatic tissues by professional Ag-presenting cells (APCs) presenting their specific antigen on major histocompatibility complex (MHC) class II molecules and providing costimulatory signals, for example in the context of an infection with microbial pathogens. Activated CD4^+^ T cells proliferate and differentiate into specific Th subsets, which will mount distinct immune responses directed against specific pathogens [8]. After the infectious pathogen has been cleared, the majority of effector Th cells will undergo apoptosis, while the remaining cells contribute to the CD4^+^ memory T cell pool [9]. The importance of memory T cell generation centers on the ability to provide a faster and augmented immune response upon secondary exposure to previously encountered microbial pathogens. The ability of memory T cells to respond faster and more efficiently is based on several essential characteristics, which render them superior in their ability to alter the outcome of infections. First, memory T cells have a lower activation threshold and are less co-stimulation dependent [10]. Therefore, upon re-challenge, memory T cells will generate robust effector responses more effectively and faster as compared with a primary T cell response [11,12,13]. Similar to the effector response generated by recently-activated naive T cells, the effector response of memory T cells is dependent on the nature and context of their encounter with their cognate antigen, for example provided by cues such as cytokines in the microenvironment [14]. Second, the frequencies of Ag-experienced memory T cells are much higher than those of naïve T cells during a primary immune response [14,15,16]. The higher frequencies of Ag-specific memory T cells increase their likelihood to encounter their cognate antigen faster upon re-infection, and to more rapidly generate a larger effector T cell pool [17,18]. Third, unlike naïve T cells, which circulate between secondary lymphatic tissues (i.e., lymph nodes) and blood, memory T cells circulate between lymphatic, blood, and peripheral tissues (e.g., lungs, gut, or skin) [19,20]. This allows memory T cells to directly and rapidly respond to the presence of pathogens in peripheral tissues. More recently a subset of non-circulating and tissue-homing memory T cells was identified and termed T_RM_ [21,22,23]. Both CD8^+^ and CD4^+^ T_RM_ have been described [reviewed in 24]. T_RM_ cells migrate to specific peripheral tissues locations, including the skin, liver, and lungs, and take up permanent residency. Importantly, the strategic location of T_RM_ at many barrier sites such as the skin and mucosal tissues, where pathogens preferentially seek access, enhances the likelihood that they rapidly encounter pathogens upon infection. CD8^+^ T_RM_ have been reviewed elsewhere [24,25,26] and this review will focus on CD4^+^ T_RM_.

Taken together, the unique characteristics of memory T cells enhance their ability for in situ immune surveillance, increase their likelihood for faster encounter of pathogens at the site of infection, and facilitate the generation of more rapid and generally superior effector responses.

## 2. Memory T Cell Development

### 2.1. Development Pathways

**Figure 1:**
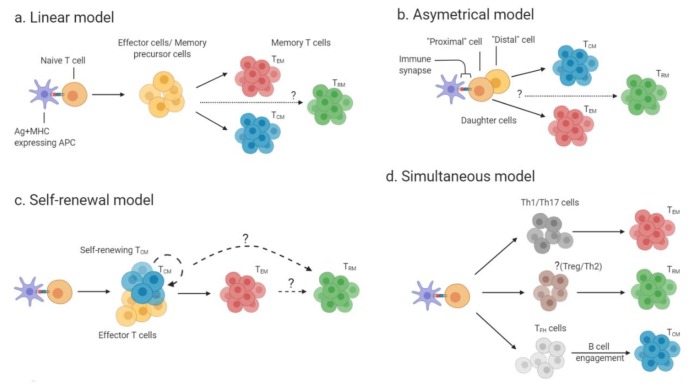
Developmental models of memory T cells: (**a**) In the linear model, effector T cells or memory precursor cells (yellow) are generated following activation of naïve T cell by antigen-presenting cells (APCs) presenting peptide on major histocompatibility complex (MHC) molecules. The intermediate effectors or memory precursors give rise to mature effector memory T cells (T_EM)_ (red) and central memory T cells (T_CM)_ (blue). It remains to be answered if the intermediate effectors/precursors also give rise to tissue-resident memory T cells (T_RM_) (**b**) In the asymmetrical model, the proximal daughter cells to the immune-synapse (naïve T cell- T cell receptor (TCR) + peptide and MHC-APC) develop into T_EM_, while the distal daughter cells develop into T_CM_. It is currently unknown which cells give rise to T_RM_ (green). (**c**) In the self-renewal model self-renewing effector T cells or T_CM_ are generated from naïve T cells. These self-renewing cells can then give rise to T_EM_ cells. It is unresolved if T_RM_ are generated from self-renewing T_CM_/effector cells or from T_EM_. (**d**) In the simultaneous model naïve T cells first differentiate into different T cell subsets. T cell subsets give rise to different memory subsets as follows: Th1 and Th17 cells (dark gray) generate T_EM_, while T_FH_ cells (light gray) generate T_CM_. The T helper cell subset(s) that generate T_RM_ has not yet been identified.

The developmental pathways of memory T cells are still not fully understood, and there are some controversies as to the different models proposed (reviewed in [27]), and compelling evidence exists for each of the models. The models are shown in Figure 1 and are summarized below.

The linear model of memory T cell development (Figure 1a) suggests that during the contraction phase of a primary T cell immune response, the surviving effector T cells differentiate into T_EM_, which will then give rise to T_CM_ [27,28]. In contrast, the asymmetrical model of memory T cell development (Figure 1b), also called the bifurcative model, suggests that two daughter cells of the same T cell clone can undergo different fates: the daughter cell proximal to the immunological synapse can give rise to both terminally-differentiated effector cells and T_EM_, while the distal daughter cell gives rise to T_CM_ [29]. A third model (Figure 1c, self-renewal model) proposes that naïve T cells first give rise to either self-renewing T_CM_ or effector T cells, and that those can further differentiate to T_EM_, which give rise to terminally-differentiated effector cells in non-lymphoid tissues [27]. Pepper and Jenkins suggested an additional model for the generation of memory CD4^+^ T cells. In this “simultaneous” model (Figure 1d), the effector T cell subset (e.g., Th1 or Th2) determines the fate of the generated memory T cell subset. For instance, Th1 cell or Th17 cell subsets will give rise to T_EM_, whereas T follicular helper (T_FH_) cell subset will generate T_CM_ upon help from B cells [30].

### 2.2. Role of T Cell Receptor Signaling Strength and Precursor Frequencies for Memory T Cell Development

Central to memory T cell development are the roles of T cell receptor (TCR) signaling strength and T cell precursor frequencies. The TCR signaling strength is determined by the affinity of the TCR for Ag and MHC molecules, the density of antigen presented on APCs, and the duration of the TCR interaction with peptide-loaded MHC [31,32]. The degree of these parameters affects TCR-dependent biochemical pathways which result in changes to T cell transcriptional profiles, to either promote or inhibit memory T cell generation [33,34]. This appears to be regulated via differences in the ratios of several transcription factors, including Bcl-6, Blimp-1, Eomes, and T-bet [33,35,36], their upstream regulators nuclear factor of activated T-cells (NFAT) and nuclear factor kappa-light-chain-enhancer of activated B cells (NF-κB), or by directly affecting the expression of cytokines and cytokine receptors, such as IL-2 and the IL-2 receptor alpha chain (CD25), which synergize with TCR signaling to affect memory-cell development [35,37,38]. For memory CD8^+^ T cells, it is generally accepted that TCR signaling strength inversely correlates with memory-cell formation [33]. However, for the generation of memory CD4^+^ T cells the data are less clear, as described below [38,39,40,41].

CD4^+^ T cells have been shown in several systems to compete for Ag, and a higher TCR affinity for Ag may affect the access of T cells to Ag [42,43,44]. Thus, TCRs with higher affinity for Ag are likely to promote greater expansion of Ag-specific T cells, and thereby increase the reservoir of T cells that could potentially enter the memory T cell pool. Furthermore, stable and sustained interaction with antigens is a critical determining factor for promoting the differentiation of memory CD4^+^ T cells during acute viral infection [45]. In contrast, low affinity TCRs for MHC plus Ag results in T cell memory with shorter lifespan and with impaired secondary responses upon re-challenge [46,47]. Not only may TCR affinity for MHC and Ag influence the generation of memory T cells, but it may also affect their recruitment into different memory T cell subsets and their lifespan. McKinstry et al. reported that CD4^+^ T cells require signals from MHC class-II molecules and CD70 during the effector phase of an immune response, and are dependent on IL-2 signaling in order to generate long-lived memory cells [48]. It has been proposed that T_CM_ may require relatively stronger TCR stimuli for their generation, while relatively weaker antigenic stimuli may generate T_EM_. Consistent with this notion, naïve CD4^+^ T cells require prolonged exposure to antigen during the expansion phase to generate T cell memory [39].

However, memory T cell formation is not only dependent on TCR affinity/antigen accessibility, but also on the frequencies of naïve T cell precursors [49]. The naïve T cell precursor frequency is defined by the fraction of naive T cells capable of responding to a specific antigenic epitope and to enter the effector/memory Ag-specific T cell pool. For example, excessively high precursor frequencies of Ag-specific T cells seem to have a negative impact on memory T cell formation. Accordingly, high precursor frequencies of adoptively-transferred TCR-transgenic CD4^+^ T cells reduced the proliferation and differentiation of these cells upon infection, and thereby resulted in impaired memory T cell formation [50]. This observation is further supported by the inverse relationship between T cell precursor frequencies and the survival of both naïve and memory CD4^+^ T cells [51]. Similarly, Blair and Lefrançois showed that transfer of high precursor frequencies of TCR-transgenic naïve CD4^+^ T cells resulted in a lack of T cell memory, which was linked to impaired effector T cell induction, reduced proliferation, and cytokine production [52]. Importantly, this phenomenon was independent of IL-7R expression by the responding memory T cells. Additionally, they showed that competition for antigen during CD4^+^ T cell priming is a major confounding factor for the development of the memory T cell pool [52]. However, in these studies, during the initial priming of naïve CD4^+^ T cells, the availability of Ag rather than the frequency of precursor cells per se appeared to be pivotal for the formation of CD4^+^ T cell memory. Nevertheless, Ag persistence negatively affects the function of CD4^+^ memory T lymphocytes and impairs their ability to produce effector cytokines, perhaps by promoting the generation of T_CM_ rather than T_EM_ [53].

### 2.3. Role of Transcription Factors and Cytokine Signaling for Memory T Cell Development

T-bet, the master-regulator of Th1 cells, acts in an expression-level-dependent manner to regulate formation of memory and effector CD4^+^ T cells, such that high expression of T-bet promotes terminal effector cells, while intermediate to low expression promotes memory development [36]. Furthermore, T-bet appears to regulate the generation of memory T cell subsets from effector cells: T-bet^low^ effector cells express the chemokine receptor (CCR) 7 and give rise to T_CM_ cells, whereas T-bet^high^ effector cells rapidly produce interferon (IFN)-γ, lack CCR7 expression and give rise to T_EM_ cells [54]. Similar to CD4^+^ memory T cells, high levels of T-bet expression by CD8^+^ effector T cells generate short-lived memory cells and favor terminally-differentiated effector cells, while low expression levels generate long-lived memory cells [55]. Along these lines, the cytokine IFN-γ, which induces T-bet expression in a signal transducer and activator of transcription (STAT) 1-dependent manner, has been shown to enhance the development of memory CD4^+^ T cells generated from both naïve and effector cells [47,56,57]. Additionally, IFN-γ producing effector cells give rise to long-lived T_CM_ and T_EM_ [58]. Thus, it appears that effector cytokines present during the primary response, and in particular IFN-γ, may tip the balance in favor of the generation of specific memory T cell subsets. Moreover, since T_EM_ cells are generated from progenitor cells which express a master regulator (e.g., T-bet for Th1), it appears that T_EM_ cells maintain lineage integrity, whereas T_CM_ cells that are generated from progenitor cells that lack (or express at low levels) a master regulator show more lineage plasticity and can generate different effector responses upon re-challenge [30,54]. Similarly, IL-12 induces the expression of T-bet in a STAT4-dependent manner in CD4^+^ T cells [59], and IL-12 promotes development of hepatitis B virus (HBV)-specific CD8^+^ T_EM_ cells [60]. Along these lines, although no data exists for the role of IL-12 in development of CD4^+^ memory T cells, mice lacking both STAT4 and T-bet have marginally reduced virus-specific CD4^+^ memory T cell frequencies as compared with mice lacking either one of these transcription factors (STAT4 or T-bet) [61]. These data suggest that early activation of STAT4 and T-bet may not be required for development of CD4^+^ T cell memory; however, their expression may affect the quality of memory responses during recall and the type (subset) of memory generated. Although CD4^+^ T_RM_ were reported to express low levels of T-bet [62], suggesting that this pathway is dispensable for CD4 T_RM_ maintenance, future work should investigate the role of T-bet-promoting cytokines for regulating CD4^+^ T_RM_ development, since this has not yet been fully resolved.

IL-2, via regulation of Bcl-6 expression, also plays fundamental roles in memory CD4 T cell development [63,64,65]. Current dogma suggests that naïve CD4^+^ T cells require high levels of IL-2 to differentiate into memory precursor cells; however, these precursor cells are then dependent on low levels of IL-2 to develop into long-lived memory cells. IL-2 signals sustain the expression of STAT5 and Bcl-6, and promote expression of IL-7 receptor, thereby enabling the survival and maturation of CD4^+^ memory T cells [48,66]. Interestingly, however, IL-2-deficient memory CD4^+^ T cells generate a more vigorous and effective recall response against influenza virus than wild-type memory cells that produce IL-2 [67], suggesting that IL-2 and Bcl-6 may have regulatory or inhibitory roles once Ag-specific T cell memory has established, perhaps to prevent immunopathology during chronic antigen exposure. Of note, IL-2, IL-7, and IL-15, also known as common-gamma chain receptor cytokines, play key roles in memory (as well as in naïve and effector) CD4^+^ and CD8^+^ T cell development, homeostasis, and maintenance [68,69,70]. The functions of these cytokines in CD4^+^ T cell memory remain somewhat controversial, and this has been reviewed elsewhere [10,68].

**Figure 2:**
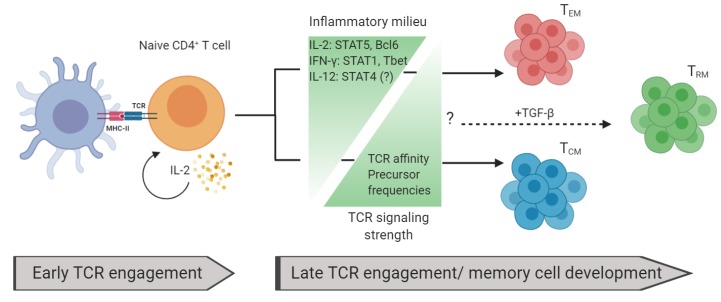
Factors influencing memory T cell development: Naïve T cells are activated by peptide and MHC via TCR. High levels of interleukin (IL)-2 are critical during early TCR engagement for memory-cell development. The memory T cell development fate is dependent on the inflammatory milieu and TCR signaling strength. Increased levels of inflammatory signals favor T_EM_ generation and decreased levels of these signals favor T_CM_. Conversely, increased levels of TCR affinity and precursor frequencies favor T_CM_ development while decreased levels favor T_EM_. TGF-β is important for generating T_RM._ How other inflammatory signals and TCR signaling strength affect T_RM_ generation remains unresolved.

Shown in Figure 2 is the integration of TCR signaling- and inflammation-dependent factors influencing memory T cell development.

Naturally, models of T cell memory development became more complex with the discovery of T_RM_, and there are still many open questions, in particular for the generation of CD4^+^ T_RM_ [15,71]. Nevertheless, it is widely accepted that T_RM_ require transforming growth factor (TGF)-β for their generation, which can be produced by a variety of cells in tissues, including fibroblasts, epithelial cells, keratinocytes, and enterocytes [72,73]. While CD8^+^ T_RM_ have been at the center of research in this area, as much as 20% of CD4^+^ T cells in certain tissues, such as the intestinal epithelium, also express CD103, the prototypical marker for tissue-resident memory cells [74]. TGF-β induces the expression of CD103 by T_RM_ precursors and promotes their entry and retention in epithelial sites [72,73]. Moreover, a sizable fraction of CD4^+^ T_RM_ express Foxp3^+^, a key transcription factor of regulatory T cells, which is induced by TGF-β signaling [75]. Therefore, these data suggest that TGF-β is a pivotal factor in CD4^+^ T_RM_ generation and that CD4^+^ T_RM_ may assume regulatory functions [76]. Future investigations will elucidate the mechanisms guiding the generation of CD4^+^ T_RM_, as well as characterize their functions.

Taken together, several models have been proposed for the generation of memory T lymphocytes. It is conceivable that more than one of these models contribute to memory T cell development in vivo, and may be influenced by factors such as TCR signal strength and inflammatory conditions in the microenvironment (cytokines), and in addition, costimulatory signals (e.g., CD28 family) [77] and other environmental cues, such as chemokines and even the microbiota from the tissue in which a T cell is activated [78,79,80].

## 3. Memory T Cell Subsets and Function

### 3.1. Memory T Cell Phenotype

**Figure 3:**
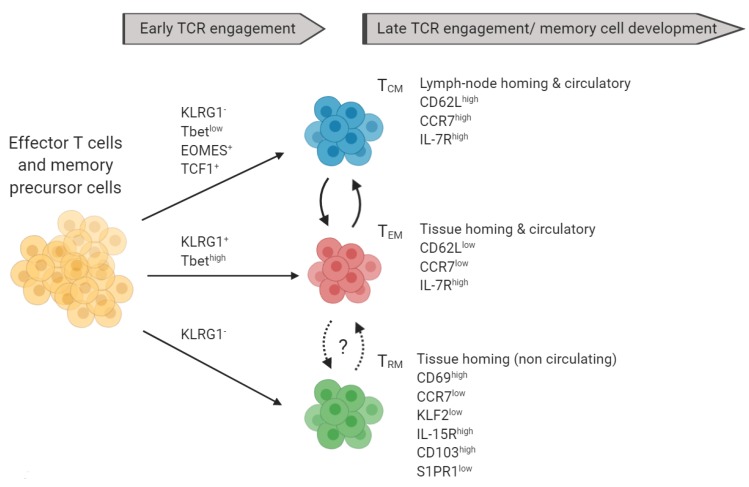
Markers of memory T cell subsets and their precursors: Terminally differentiated T_CM_ (blue) and T_EM_ (red) can be distinguished based on the expression of CD62L, CCR7, IL7R, and other markers not shown. T_RM_ can be further characterized based on the expression of KLF2, IL-15R, CD103, and S1PR1. T_CM_ can give rise to T_EM_ and vice versa. *Recent data suggested that T_RM_ can re-enter the circulation.

Memory T cells are generally subdivided into three main populations: T_CM_, T_EM_, and T_RM_. Human memory T cells share many phenotypic characteristics with other species such as mouse. The phenotype of the different memory T cell subsets is discussed below and summarized in Figure 3.

Human memory T cells can be distinguished from naïve T cells by expression changes of the CD45 isoforms and increased capacity for effector cytokine production upon antigen recall [81,82,83]. Human memory T cells were initially distinguished from naïve T cells by being CD45RO^+^ and CD45RA^−^, whereas naïve T cells show the CD45RO^−^ CD45RA^+^ phenotype [84,85,86]. Sallusto et al. subsequently showed that CD45RA^−^ memory T cells could be further divided into two subpopulations based on the expression of the chemokine receptor CCR7 [28]. They proposed a model in which CD45RA^−^ CCR7^+^ T cells recirculated to lymphatic tissues and termed these cells T_CM_, whereas CD45RA^−^ CCR7^−^ T cells, which remained in the immune periphery, were termed T_EM_ [28,87].

For mice, Bradley et al. showed that mouse naïve and memory T cells can be phenotypically distinguished based on the expression of CD62L (L-selectin) and CD44, and increased expression of effector cytokines upon re-challenge. Naïve cells are CD62L^+^ and CD44^low^, whereas memory cells (T_EM_) are CD62L^−^ and CD44^high^ [88,89]. Additional differences in cell surface marker expression in mouse (and human) T cells have emerged to allow classification of naïve vs. memory T cells, such as CD69 and chemokine receptors [90,91,92]. Reinhardt et al. showed that the CD4^+^ T_CM_ and T_EM_ paradigm also translates to mice, and that these memory-cell subsets can be distinguished based on the expression of CD62L and CCR7 [87,93,94]. Thus, both mouse and human T_CM_ are CD62L^+^ CCR7^+^, while T_EM_ are CD62L^−^ CCR7^−^ [95]. Therefore, in addition to classifying memory vs. naïve T cells based on CD45 isoforms, both human and mouse naïve T cells can be characterized by a CD44^low^ CCR7^+^ CD62L^high^ phenotype, whereas T_CM_ are CD44^high^ CCR7^+^ CD62L^high^, and T_EM_ cells are CD44^high^ CCR7^−^ CD62L^low^ [96,97]. Unlike human T cells, mouse naïve and memory T cells cannot be distinguished based on the expression of CD45 isoforms [98].

Recently, Lefrançois and colleagues described an additional memory T cell subset, termed T_RM_ [99]. This subset resides in peripheral tissues during or after infectious encounters and does not recirculate between blood, lymphatics, or other peripheral tissues, as do T_CM_ or T_EM_ [100,101,102]. T_RM_ constitutively express CD69, CD103 (integrin alpha E, ITGAE; most prominent in CD8^+^ T_RM_), and S1PR1 (sphingosine-1-phosphate receptor 1), but they do not express CCR7 or CD62L [23,24,102]. The expression of CD69, CD103, and S1PR1 by T_RM_, together with the absence of CCR7 expression, promotes their tissue homing and impedes their tissue egress [24,100,101]. Interestingly, the expression of CD69 and CD103 by T_RM_ is independent of TCR signaling and Ag persistence, and may be dependent on constitutive signaling of “alarm cytokines” such as IL-33 and type I interferons [103,104]. Human T_RM_ are also CD45RO^+^ CD45RA^−^ [105]. Interestingly, Kumar et al. recently identified a core transcriptional profile for human CD4^+^ and CD8^+^ T_RM_ at various sites that shows increased expression of specific adhesion molecules (such as CD103) and production of both pro-inflammatory and regulatory cytokines and chemokines (such as IL-2 and IL-10) [106].

Recently, the dogma of T_RM_ as a non-circulating subset was challenged by identifying a population of circulating CD4^+^ T_RM_ in human blood, which was designated as “ex-T_RM_” [107]. These CD4^+^ ex-T_RM_ express the skin homing and retention glycan cutaneous lymphocyte-associated antigen (CLA), have similar phenotypic and transcriptional attributes as skin resident CD4^+^ CD103^+^ CLA^+^ T_RM_, and share a clonal origin with CD4^+^ CD103^+^ CLA^+^ T_RM_ in the skin, based on TCR sequencing [108]. Thus, these new data suggest that CD4^+^ T_RM_ may reside in tissues for prolonged periods of time, but possibly not for all of their lifespan.

### 3.2. Memory T Cell Subset Function

All memory CD4^+^ T cell subsets play a pivotal role in defending against pathogens [109,110,111]. However, their individual contributions vary, which is to some degree a function of their respective migratory properties and tissue homing [112]. Along these lines, the expression of CD62L and CCR7 by T_CM_ endows them with the ability to migrate and home to secondary lymphatic tissues, and thereby facilitates immune surveillance of antigens collected via lymphatic drainage or dendritic cells (DCs) from peripheral tissues [113,114]. The frequencies of Ag-specific T_CM_ are as much as thousand-fold increased as compared with Ag-specific naïve T cells, and thereby they are able to rapidly generate a robust effector T cell pool upon secondary encounter of cognate antigens [16]. Moreover, while T_CM_ show a lower capacity for the production of some cytokines, such as IFN-γ and IL-4, as compared with T_EM_, they produce more IL-2 and have an overall greater capacity for proliferation [93,115]. Thus, T_CM_ have a higher likelihood to become activated by APCs in lymphatic tissues upon re-infection with a previously encountered pathogen to provide a stronger and more rapid response and proliferate rapidly to generate a large pool of pathogen-specific effector T cells. Furthermore, CD4^+^ T_CM_ provide superior B cell help, which results in faster B cell expansion, more rapid class switching, and increased antibody production [89,116]. Indeed, CD4^+^ T_CM_ with T_FH_ cell phenotype persist in germinal centers of draining lymph nodes following vaccination to regulate memory B cell development and maintenance and support rapid generation of long-lived plasma cells upon re-exposure to antigen [117,118].

In contrast to T_CM_, T_EM_ preferentially recirculate between blood and peripheral tissues. As indicated by their designation, T_EM_ rapidly exhibit effector functions, such as the production of cytokines upon activation. Interestingly, IL-1β promotes effector-cytokine production, such as IL-17 and IFN-γ, by CD4 T_EM_ cells by stabilizing the cytokine transcripts upon Ag-encounter [119]. T_EM_ have a longer lifespan as compared with effector T cells and provide a readily-available pool for effector T cells in the immune periphery [113]. Thus, T_EM_ can quickly supply Ag-specific effector T cells in peripheral tissues when the need arises, as compared with the longer time required for the differentiation of naïve T cells into effector T cells.

### 3.3. CD4^+^ T_RM_ Subset Function

CD4^+^ T_RM_, which reside in peripheral tissues, function as the first line of defense at these sites together with T_EM_. However, T_RM_ exhibit some unique functions different from those of T_EM_, and in some cases, show more vigorous responses during secondary Ag-encounter [120,121,122,123]. For instance, lung-resident memory CD4^+^ T_RM_ cells provide optimal protection against secondary respiratory viral challenge with influenza virus, whereas protection provided by influenza-specific circulating memory CD4^+^ T cells is weaker despite their ability to expand and migrate to the lungs upon infection with the same pathogens [121]. Likewise, optimal protection against *Chlamydia* infection is dependent on the generation of mucosa (genital tract) homing CD4^+^ T_RM_, while protection provided by circulating memory T cells is less effective [124] suggesting that mucosal CD4^+^ T_RM_ are critical for optimal protection against pathogens entering via the mucosal entry sites. Of note, tumor-homing CD4^+^ T_RM_ are more potent producers of TNF and IFN-γ compared with other tumor infiltrating T cells [125]. Additionally, CD4^+^ T_RM_ directed against certain pathogens emerge and persist in peripheral tissues following infection, such as influenza-virus-specific CD4^+^ T_RM_ in the lungs and *Leishmania-*specific memory CD4^+^ T_RM_ in the skin [126,127]. These CD4^+^ T_RM_ cells rapidly produce effector cytokines such as IFN-γ and IL-17 upon re-challenge [126,127]. Using a new strategy for mucosal vaccination, Stary et al. showed that IFN-γ producing CD4^+^ T_RM_ are pivotal for protection against *Chlamydia trachomatis* [124,128]. Similar findings have been reported for genital tract herpes simplex virus (HSV) vaccination [120], and gastric subserous vaccination with *Helicobacter pylori* vaccine [129]. Therefore, identification of mechanisms which promote the generation and retention of CD4^+^ T_RM_ should be further explored for development of more effective vaccines against a range of human pathogens [130]. Of note, female lower genital tract CD4^+^ T_RM_ were identified to serve as primary targets of HIV infection and persistence, thus providing an HIV cellular sanctuary [131]. Thus, HIV treatment strategies and vaccines may consider targeting T_RM_ [131].

The mechanisms by which CD4^+^ T_RM_ provide enhanced protection is an area of intense research, and some evidence suggests that they may differ from those used by circulating effector/memory CD4^+^ T cells. Along this line, CD4^+^ T_RM_ provide rapid protection by promoting the recruitment of immune cells into the affected tissues [121,122,132,133,134]. In addition, CD4^+^ T_RM_ are important for the maintenance, distribution, and homing of CD8^+^ T_RM_ in situ [135,136]. Since it was shown that CD4^+^ T cells can foster the development of lung CD8^+^ T_RM_ cells during infection with influenza virus [137], it is conceivable that CD4^+^ T_RM_ may also contribute to the generation of CD8^+^ T_RM._ Interestingly, CD4^+^ T_RM_ outnumber CD8^+^ T_RM_ in many tissues [23,123], suggesting a critical role for CD4^+^ T_RM_ in tissue-specific immunity and barrier function. For instance, approximately 70% to 85% of total T_RM_ in the human skin are CD4^+^ cells.

Mechanistically, CD4^+^ T_RM_ cells in the skin proliferate more extensively and produce significantly higher levels of IFN-γ, TNF, and IL-22 (and to a lesser extent IL-17 and IL-4) as compared with circulating memory CD4^+^ T cells [123]. In fact, immunosurveillance of non-lymphoid tissues is orchestrated by CD4^+^ T_RM_ cells rather than by CD8^+^ T_RM_ [138]. Notably, CD4^+^ T_RM_ share overlapping transcriptional, phenotypic, and location-specific functional properties with CD8^+^ T_RM_ and orchestrate local recall responses [138]. In contrast to CD8^+^ T_RM_, the human skin is populated with CD4^+^ T_RM_ which are either CD103^+^ and reside primarily in the epidermis, or CD103^-^ which mainly reside in the dermis [123]. Interestingly, CD103^+^ CD4^+^ T_RM_ in skin show lower proliferative capacity but increased effector function as compared with CD103^-^ CD4^+^ T_RM_, independent of their location in the dermis or epidermis [123]. These data suggest that CD103^+^ and CD103^-^ CD4^+^ T_RM_ cells encompass unique functional attributes in which CD103^+^ T_RM_ cells provide robust effector responses (cytokine production), while CD103^-^ T_RM_ cells proliferate extensively to supply the Ag-specific CD4^+^ T_RM_ cell pool. Future studies should investigate the cross-regulation between these two populations and whether CD103^−^ CD4^+^ T_RM_ can give rise to CD103^+^ T_RM_ cells or vice versa. Finally, the generation and retention of skin CD4^+^ T_RM_ was shown to be dependent on skin-resident CD8^+^ T cells or CD11b^+^ skin-resident macrophages [139], adding to the complexity in this system.

In addition to providing enhanced tissue protection, CD4^+^ T_RM_ have also been implicated in undesired immunopathology of inflammatory diseases and they may contribute to the persistence of inflammatory cells and chronic inflammation in the affected tissues [140,141,142]. Nevertheless, approximately 10% of CD4^+^ T_RM_ express the transcription factor Foxp3 and are thought to have regulatory functions [143]. In this context, Foxp3^+^ CD4^+^ T cells enter and reside in the skin during the neonatal period and mediate tolerance to commensal, non-pathogenic microbes [144]. Therefore, it will be critical to elucidate the mechanisms of CD4^+^ T_RM_ developmental pathways, generation and maintenance, and their intersection with anti-microbial, regulatory, or pathologic functions to elicit optimal protection, while avoiding tissue damage.

In summary, memory CD4^+^ T cell responses and their unique functional attributes provide critical contributions to protection against microbial pathogens [111]. These include increased cytokine production, regulation of innate immune cell functions, mobilization of immune cells to sites of infection, providing B cell help, and enhancing cytotoxic T cell responses [111]. Robust and rapid local responses are provided at entry sites of infection by CD4^+^ T_RM_, which tailor immune-responses to specific tissues and the local microenvironment by providing local cues, mediating the rapid recruitment of other immune cells, and by regulating and maintaining other tissue-resident cells, including CD8^+^ T_RM_.

## 4. Memory CD4^+^ T Cells in Autoimmunity

Memory CD4^+^ T cells are of great interest in the context of autoimmune diseases because of their long-lived nature, efficient responses to antigens, and unique potential to mediate recurring autoimmune responses. However, until now, T cell memory has been more extensively investigated in the context of infectious diseases and its role in autoimmune diseases is not fully elucidated. Here, we will summarize and discuss some of the most pertinent findings on memory CD4^+^ T cells in autoimmune diseases, with special focus on multiple sclerosis (MS) and its animal model experimental autoimmune encephalomyelitis (EAE).

### 4.1. Persistence of Autoreactive Memory T Cells in Autoimmune Diseases and the Role of Immunoscenescence

Autoreactive memory CD4^+^ T cells have been studied in patients in several autoimmune disease conditions [145,146]. For instance, patients with MS and psoriasis show increased numbers of memory CD4^+^ T cells as compared with healthy individuals, suggesting that memory CD4^+^ T cell are critical mediators of autoimmune disease [147]. Subsequent work in animal models of autoimmune diseases further highlighted the roles which memory CD4^+^ T cell play in promoting autoimmunity. Along these lines, adoptive transfer of autoreactive memory CD4^+^ T cells is sufficient to induce disease, for example in animal models of MS, diabetes, and uveoretinitis [148,149,150]. However, while autoreactive CD4^+^ memory T cells are sufficient to induce autoimmune pathology, the context that these cells are transferred into is important. For example, in an elegant study in EAE, neonatal mice were injected with myelin basic protein (MBP)-specific CD4^+^ T cells to allow the generation of MBP-specific memory CD4^+^ T cells prior to adulthood [151]. Importantly, these animals remained healthy despite the presence of memory CD4^+^ T cells in lymphoid tissues when they reached adulthood. However, mice that had received MBP-specific CD4^+^ T cells developed earlier and had more severe EAE disease when they were immunized with MBP in complete Freud’s adjuvant (CFA) as compared with mice that received ovalbumin (OVA)-specific CD4^+^ T cells. These data suggest that memory CD4^+^ T cells that develop at early ages (prior to adulthood) may have regulatory functions, or that autoreactive memory T cells can form and persist in healthy individuals but may require additional events in order to become activated and induce disease. Nevertheless, these data demonstrate that autoreactive CD4^+^ memory T cells generate a more vigorous response upon exposure to autoantigen as compared with Ag-inexperienced naïve autoreactive T cells. These data also suggest that the re-activation of autoimmune memory T cells requires a lower Ag threshold than that of naïve T cells, similar to pathogen-reactive memory T cells.

Along these lines, a critical unresolved question for future studies is how T cell senescence and aging affects memory T cell functions. The effect of aging on CD4^+^ naïve and memory T cells in the context of infectious diseases has been studied and extensively reviewed elsewhere [152,153,154,155]. Immunoscenescence (i.e., ageing of the immune system) is characterized by a decline of adoptive and innate immune cell functions, including of CD4^+^ T cells [152,156]. However, aging is also associated with autoimmune phenomena, and certain autoimmune disease conditions are more frequently observed in elderly individuals [157]. Moreover, aging and T cell immunoscenescence has been associated with MS and EAE [158]), as well as with other autoimmune diseases [159,160]). Furthermore, treatment efficacy and development of progressive multifocal leukoencephalopathy in MS patients has been linked and associated with immunoscenescence [161,162,163]. As the role of the aging immune system in infectious diseases and autoimmunity appears somewhat contradictory [160], an interesting question is how does the inflammatory environment during aging/immunoscenescence contribute to and modulate autoimmune memory? Along these lines, a recent interesting study showed that aging promotes the development and accumulation of extreme pro-inflammatory cytotoxic CD4^+^ T cells, as well as anti-inflammatory regulatory T cells (Tregs) (~30% of the total T cell pool) [164]. How these findings affect autoreactive memory T cells will be an interesting question for further research. Furthermore, many pressing questions remain to be further explored, for instance, which antigens these cells recognize and/or their clonotypes both in healthy individuals as well as in autoimmune disease, what are the mechanisms that maintain or revoke the activation/quiescence of these cells, and what is their relationship to memory T cells? Taken together, immunoscenescence may have important implications for the approach to treating autoimmune diseases in the elderly.

### 4.2. Role of Autoantigen for Memory T Cells

A question central to autoimmune memory is which autoantigens the memory CD4^+^ T cells recognize in human autoimmune diseases. The answer to this question could provide insights into mechanisms that promote escape from immune tolerance. For instance, rheumatoid arthritis (RA) patients exhibit memory CD4^+^ T cells specific for various citrullinated antigens, including citrullinated aggrecan and citrullinated vimentin, which correspond to autoantibodies directed against citrullinated antigens and proteins in these patients [165,166,167]. Furthermore, CD4^+^ memory cells with a Th17 phenotype and specific for a citrullinated vimentin epitope expanded more significantly in RA patients with active disease and significantly decreased upon anti-TNF treatment [168]. Additionally, memory CD4^+^ T cells recognizing glycosylated type II collagen peptides have been identified in RA patients [169]. Together, these data suggest that post-translationally modified autoantigens, and particularly citrullinated autoantigens, may be critical to drive RA progression and increase the number of autoreactive memory T cell clones. Memory CD4^+^ T cells from MS patients have been reported to recognize several neuroantigens, particularly myelin antigens, including myelin oligodendrocyte glycoprotein (MOG) and MBP [170,171], and T cell responses against these autoantigens are pathogenic in its animal model EAE [151]. It remains to be determined whether modifications of myelin antigens also play a role in MS patients, similar to RA, and whether this results in generation of autoreactive memory CD4^+^ T cells. This may provide important clues for the role of autoreactive memory T cells in disease etiology and progression. Taken together, strong evidence supports a key role for memory CD4^+^ T cells in the pathogenesis of autoimmune diseases. Important remaining questions will center on the mechanisms that activate, sustain, and regulate these autoreactive T cells.

### 4.3. Autoreactive Memory T Cells with Th17 Cell Phenotype

Th17 cells are key drivers of many chronic autoimmune disease conditions, including MS, RA, and psoriasis [2,172,173]. Thus, memory T cells with a Th17 cell phenotype have been reported in many of these conditions. Th17 cells can give rise to self-renewing CD4^+^ T_EM_ that maintain their Th17 cell phenotype [174]. Moreover, CD4^+^ T_CM_ with Th1 and Th17 phenotype were reported as selectively increased in blood of MS patients and to correlate with disease severity. Interestingly, the transcriptional profile of blood Th1 T_CM_ and Th17 T_CM_ strongly resembled conventional effector Th17 cells (and not Th1 cells) with more pathogenic features. The cerebrospinal fluid (CSF) of these patients contained mainly CXCR3-expressing Th1/Th17 T_CM_ cells. However, CSF Th17 T_CM_ cells of MS patients reacted strongly to myelin-derived self-antigens (including MOG and MBP), while Th1 cells responded consistently only to virus antigens (such as Epstein–Barr virus). Additionally, the CSF Th1 T_CM_ and Th17 T_CM_ from MS patients had the capacity to produce high levels of pathogenic cytokines upon activation, including IFN-γ, IL-17, GM-CSF, and IL-22 [170]. Moreover, IL-23, which is essential for terminal differentiation of pathogenic Th17 cells [175], has been proposed to regulate memory Th17 cell generation and function. In support of this view, IL-23 drives the proliferation and expansion of memory Th17 cells from MS patients and promotes expression of IL-17 and IFN-γ in these cells [176]. Similarly, IL-23 signaling is critical for development and proliferation of memory Th17 cells in EAE [177], and memory CD4^+^ T cells in EAE mice proliferate more and produce more IFN-γ but less IL-17 as compared with effector T cells [148]. Furthermore, a population of gut resident CD161^+^ Th17 cells was identified in patients with Crohn’s disease. These gut-homing memory Th17 cells expressed high levels of pro-inflammatory cytokines, including IL-17, IL-22, and IFN-γ upon re-activation with αCD3 and αCD28 in presence of IL-1β and IL-23 [178]. Moreover, in an animal model of colitis, IL-23 promotes the proliferation of memory CD4^+^ T cells and increases the expression of IFN-γ and IL-17 to promote inflammation [179]. Thought-provokingly, IL-21, a cytokine that is expressed by Th17 cells and enhances their development and expansion in an autocrine fashion [180,181], was reported to inhibit de novo generation of pathogenic Th17 (and Th1) effector T cells from IL-21-expressing T_CM_ cells. These data indicate a potentially protective role for IL-21^+^ T_CM_ in the context of autoimmunity [182].

### 4.4. Autoreactive T_CM_ and T_EM_ Subsets and Disease-Modifying Therapies

A critical remaining question centers on the functional attributes of the different memory CD4^+^ T cell subsets (i.e., T_CM_, T_EM,_ and T_RM_) in promoting autoimmune disease. Although both autoantigen- specific CD4^+^ T_CM_ and T_EM_ cells have been reported, the generation of CD4^+^ T_EM_ appears more common [183]. This phenomenon is attributed to the presence of chronic autoantigen exposure, which appears to favor the development of CD4^+^ T_EM_ while hindering CD4^+^ T_CM_ cell formation, similar to chronic infection settings [174,178,183]. Along these lines, MS and systemic lupus erythematosus (SLE) patients show higher frequencies of autoreactive CD4^+^ T_EM_ and lower CD4^+^ T_CM_ in peripheral blood compared with healthy controls [183]. Similar results were reported in patients with colitis, type 1 diabetes, and other autoimmune diseases [174,178,183]. Interestingly, unlike pathogen-specific memory T cells, which are long-lived and highly proliferative, memory CD4^+^ T cells from autoimmune disease patients are more likely to undergo apoptosis and are less likely to proliferate, most notably for CD4^+^ T_CM_ [183]. These data suggest that chronic autoimmune disease conditions promote memory CD4^+^ T cell death and inhibit their proliferation and survival. Determining how differentiation and survival of different subsets of memory CD4^+^ T cells in autoimmune disease conditions are affected by factors such as cytokine milieu and the presence of autoantigens may lead to potential new avenues for treatment of disease progression and relapses.

Further implicating memory T cells in MS are the results of treating MS patients with fingolimod (also known as FTY720), an S1P receptor (S1PR) antagonist which is thought to act by downregulating the expression of S1PR1 on lymphocytes and is now approved for the treatment of relapsing MS [184,185]. Responsiveness to S1P (via S1PRs) and S1P-dependent tissue trafficking from lymphoid tissues to inflamed tissues are complex and reviewed elsewhere [186,187]. Briefly, S1P acts to promote lymph node egress by overcoming retention signals mediated by factors such as CCR7 [188]. Additionally, T_CM_ largely depend on the S1P/S1PR-axis to traffic/exit from lymphoid tissues to the blood circulation, while T_EM_ which are CCR7^low^, have already egressed to the circulation and do no-longer rely on S1P/S1PR1 [187,188]. Thus, fingolimod prevents the circulation of T_CM_ but not T_EM_ to the CNS and promotes (CCR7-mediated) T_CM_ retention in secondary lymphoid tissues [189]. T_RM_ cells do not express S1PR1 as they do not express its transcription factor KLF2 (Figure 3) [24]. Along these lines, fingolimod treatment of MS patients showed a marked reduction in blood-circulating CD4^+^ T_CM_ but not in T_EM_ [189,190]. Subsequent studies showed that fingolimod affected primarily IL-17-producing CD4^+^ T_CM_ [191]. Interestingly, fingolimod-treated relapsed MS patients showed greater percentages of CD4^+^ T_CM_ (and naïve cells) but not T_EM_, suggesting that CD4^+^ T_CM_ may be involved in promoting relapses following fingolimod treatment in MS patients [192]. Furthermore, fingolimod treatment was associated with elevated frequencies of CD56^+^ memory T cells, and increased granzyme (GZM) B, perforin, and Fas ligand expression in memory T cells in MS patients, and interestingly, this T cell phenotype was also associated with clinical relapses [192]. Additionally, Herich et al. demonstrated that CD4^+^ T_EM_ expressing high levels of CCR5 and GZMK are involved in CNS immune surveillance in healthy individuals, but that this subset was dominant in peripheral blood mononuclear cells of MS patients, and that natalizumab (anti-α4-integrin) treatment significantly decreased these cells [193]. Furthermore, the CCR5^high^ GZMK^+^ CD4^+^ T_EM_ subset shares many transcriptional features with T_RM_ and Th17 cells, suggesting that it could play a central role in CNS pathology [193].

Therapies aimed at modulating the function of autoreactive memory T cells should take advantage of the current understanding of mechanism that regulate effector T cells (Table 1). For example, are immune checkpoints similarly effective in memory T cells as compared to effector T cells? Furthermore, what is the impact of regulatory T cells on memory T cell pool and function? At least some of the mechanisms known to regulate effector T cells act differently on memory T cells, which could potentially be explored therapeutically. A better understanding of these critical mechanisms may have major implications for therapeutic intervention for autoimmune diseases, for instance by targeting the common gamma-chain-receptor cytokines (e.g., IL-7, IL-15), which greatly affect naïve and memory CD4^+^ T cell development and homeostasis in healthy individuals and infectious diseases [194,195].Thus, future work should focus on further unraveling the different mechanisms by which memory T cell subsets contribute to autoimmune inflammatory diseases, and elucidate mechanisms by which regulatory mechanisms and therapeutic drugs may affect memory T cell subset effector functions and migratory capacities.

## 5. Concluding Remarks

A better understanding of the immunobiology of CD4^+^ memory T cells in chronic autoimmune diseases is critical to develop better treatments. While it is understood that there are differences in memory T cell populations and subpopulations in autoimmune disease conditions, there are important gaps in the current understanding of how these cells develop and how the host microenvironment, including antigen exposure and cytokine milieu, affect the function and maintenance of these cells. Additionally, it remains incompletely understood if and how these cells differ from memory T cells directed against infectious pathogens in terms of activation thresholds, cytokine secretion, and long-term survival, and how regulatory mechanism apply to these cells as compared with naïve/effector T cells.

CD8^+^ and CD4^+^ T_RM_ cells were identified in human brain and in lesions of MS patients [212,213], and recent research primarily focused on the role of CD8^+^ T_RM_ cells and their contribution to autoimmune pathology in MS and EAE [212,213,214,215,216]. However, autoimmune CD4^+^ T_RM_ are not as well studied, and many critical questions remain as to their potential contribution to autoimmune disease pathology. Moreover, a better understanding of the role of autoreactive, pathogenic CD4^+^ T cells in relapses and progression of autoimmune diseases could have major therapeutic implications. Addressing these questions will be paramount to develop better treatments for CD4^+^ T cell-driven autoimmune diseases.

## Figures and Tables

**Table 1:** Regulation of memory and effector CD4^+^ T cells. Abbreviations used: programmed cell death protein 1 (PD-1), B-and T-lymphocyte attenuator (BTLA), lymphocyte-activation gene 3 (LAG-3), T-cell immunoglobulin, mucin-domain containing-3 (TIM-3), and dendritic cell (DC).

Mechanism of Regulation	Effector T Cells	Memory T Cells	Reviewed in Reference(s)
Immune checkpoints/ T cell exhaustion	PD-1 [196] BTLA [197] LAG-3 [198] TIM-3 promotes effector responses [199]	CTLA-4 [200] LAG-3 [198] TIM-3 suppresses memory differentiation [199]	[196,197,198,199,200]
Antigen persistence	Low antigen dose results in suboptimal activation High antigen and prolonged exposure results in exhaustion	Lower activation threshold Lower co-stimulation dependence may facilitate exhaustion	[53,201,202]
Regulatory T cells	Secretion of inhibitory cytokines —IL-10, TGF-β Metabolic regulation (indirectly) via modulation of DC functions	Secretion of inhibitory cytokines —IL-10, TGF-β Metabolic regulation (indirectly) via modulation of (DC) functions	[203,204,205]
Cytokines in the maintenance/development of effector and memory cells	Depends on the subset [2,206]: Th1: IL-2, IFN-γ, IL-12 Th2: IL-4, IL-2 Th17: TGF-β, IL-6, IL-1, IL-23 Tregs: TGF-β	IL-7 [207,208,209] IL-15 [210,211]	[2,206,207,208,209,210,211]
